# Circadian Rhythm Does Not Affect the miRNA Cargo of Bovine Raw Milk Extracellular Vesicles

**DOI:** 10.3390/ijms241210210

**Published:** 2023-06-16

**Authors:** Mara D. Saenz-de-Juano, Giulia Silvestrelli, Susanne E. Ulbrich

**Affiliations:** ETH Zurich, Animal Physiology, Institute of Agricultural Sciences, 8092 Zurich, Switzerland

**Keywords:** extracellular vesicles, milk, miRNA, circadian variation, small RNA-seq

## Abstract

Extracellular vesicles (EVs) and their microRNA (miRNA) cargo have been proposed as possible mammary gland health biomarkers in cattle. However, throughout the day, the biologically active milk components, such as miRNAs, may change due to the dynamic nature of milk. The current study aimed to evaluate the circadian fluctuation of milk EVs miRNA cargo to assess the feasibility of milk EVs as future biomarkers for mammary gland health management. Milk from four healthy dairy cows was collected for four consecutive days in the two daily milking sessions in the morning and the evening. The isolated EVs were heterogeneous, intact, and carried the EV protein markers CD9, CD81, and TSG101, as shown by transmission electron microscopy and western blot. The miRNA sequencing results demonstrate that the abundance of miRNA cargo in milk EVs remained stable, unlike other milk components, such as somatic cells, that changed during milking sessions. These findings indicated that the miRNA cargo within milk EVs remains stable irrespective of the time of day, suggesting their potential utility as diagnostic markers for mammary gland health.

## 1. Introduction

Mastitis is still a major issue in dairy cows and the most common reason for antimicrobial use on dairy farms [1]. The sooner the mastitis is detected, the more treatment options remain available and the fewer opportunities the disease has to progress. The most frequent diagnostic test is the routine somatic cell count (SCC) assessment, based on the increase of immune cells in the milk due to inflammation in the mammary tissue [2]. However, as soon as a high SCC is detected (>200,000 cells/mL), the infection is already established. Instead of prevention, clinical intervention remains the only treatment option. Therefore, assessing the SCC test alone does not provide direct information on mammary gland health and homeostasis. Frequent health monitoring enables prompt detection and early treatment of many pathological conditions. Automatic in-line measurement of milk composition can provide information on milk quality alterations and cow health status and be useful for managing the dairy herd [3]. Milk is routinely collected twice daily, making it a more practical supply of biomarkers than blood [4]. It is known that extracellular vesicles (EVs) are crucial in intercellular communication in both normal physiological and disease stages [5]. As they are easily accessible from all biofluids, including milk, studying EVs offers the possibility of detecting the information that cells exchange locally and at the systemic level. Milk EVs originate from the alveolar epithelium and other cells, including immune cells [6,7,8]. Recent evidence supports the biological activity of milk EV in modulating immunity-related diseases [6,7], lactation periods [9], and cell proliferation [10]. Therefore, milk EVs can be considered to reflect the physiological state of the mammary gland, explaining why there is an increasing interest in studying the cargo of milk extracellular vesicles as biomarkers of metabolism and health in dairy ruminants [11].

MicroRNAs (miRNA) are small ~22 nucleotide single-stranded RNAs that are a common and heavily studied EV cargo [12]. They have gained great biomedical interest as disease biomarkers and RNA-mediated therapies [12]. The fact that milk miRNAs are primarily expressed in the mammary gland itself rather than transferred from circulating blood makes them ideal candidates for assessing mammary gland health [13]. Differences in milk EV miRNA cargo have been detected due to mastitis [14,15,16,17], stress from relocation [18], and viral infections [19]. However, milk is a dynamic biological fluid, and circadian fluctuations have been observed in macronutrients such as fat and protein [20]. It is known that the circadian clock controls many aspects of physiology, including EV cargo in plasma EVs [21], small EVs released by tendon fibroblasts [22], and others (reviewed by [23]). In breast milk, it has been found using qPCR that hsa-miR-21-5p, hsa-miR-146-5p, hsa-let-7d-5p, hsa-let-7g-5p, and hsa-let-7a are very stable through 24 hours, while hsa-miR-16-5p had an upregulation pick in the evening [24]. Currently, there are limited studies of milk EV miRNAs and proteins as markers of metabolism and health [11], and information regarding circadian changes in bovine milk EV miRNA cargo is still missing. The current study aimed to evaluate the variance between morning and evening miRNA cargo in milk EVs from healthy cows. This information could have implications for the experimental setting, design, and analysis of milk EVs for diagnostic purposes.

## 2. Results and Discussion

### 2.1. Somatic Cell Count (SCC) Variation in Healthy Mammary Glands

Daily SCC and average SCC values are shown in Supplementary Table S1 and Figure S1. In all cows, the SCC average was below 200,000 cells/mL, indicating that the analyzed quarters were healthy [25,26]. However, we observed a fluctuation in SCC between the morning and evening. In Cow 1 and Cow 2, there was one session in which the SCC peaked above 200,000 cells/mL, but the SCC decreased in the following hours to stabilize again. This fluctuation is one disadvantage of using the SCC to diagnose subclinical mastitis because the greater the variation, the more frequent sampling is needed to be a useful diagnostic tool for management decisions [3].

### 2.2. Milk EVs Characterization

**Figure 1:**
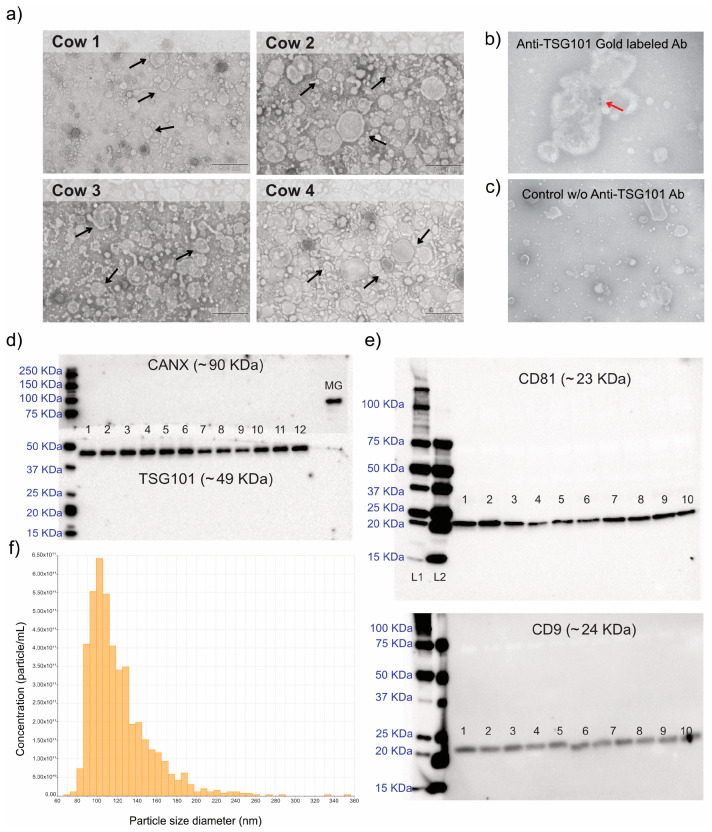
(**a**) Transmission electron microscopy (TEM) analysis of the milk EVs isolated from each cow. Black arrows show microvesicles and exosomes. (**b**) TEM image using gold-labeled anti-TSG101. The red arrow indicates the gold antibody. (**c**) TEM image showing the control using the secondary antibody but not the anti-TSg101. (**d**,**e**) Western blot analysis for CANX, TSG101, CD81, and CD9 protein markers. Pictures show full-length blots. Numbers 1 to 12 indicate different milk EV samples. MG: Mammary gland tissue. (**f**) Representative size distribution graphs from one milk EV sample measured with tunable resistive pulse sensing technology.

Milk EV characterization was performed using transmission electron microscopy (TEM), tunable resistive pulse sensing (TRPS), and western blot (WB). The EV samples were intact and heterogeneous (Figure 1a), with populations of small and large extracellular vesicles, as confirmed by TEM and TRPS (Figure 1a,f). Nanogold immunostaining of TSG101 showed the presence of the EV marker on the EVs (Figure 1b) but not in the control without a primary antibody (Figure 1c). The presence of TSG101 was also confirmed by WB (Figure 1d). Additionally, the EV protein markers CD9 and CD81 were detected by WB (Figure 1e). The absence of calnexin (Figure 1d) excluded any intracellular debris contamination from the EV isolation, and together with the TRPS results (Figure 1f), most EVs belonged to a small subtype population.

### 2.3. RNA Extraction and Data Sequencing Output

The total RNA extracted for each sample is shown in Supplementary Table S1. There was no correlation between the amount of RNA isolated and SCC (Pearson correlation test, *p*-value = 0.39). The library preparation did not work for all samples, so Cows 1 and 3 included three days of analysis (6 time points) instead of four days (8 time points). The data analysis was conducted on a local Galaxy system, utilizing the same in-house-developed pipeline previously applied for small RNA analysis [17]. Raw sequencing reads were cleaned with Trim Galore! (Version 0.4.3 by Felix Krueger) by eliminating low-quality sequences, sequencing adapters, short sequences, and PCR duplicates. The number of clean reads used for analysis for each of the 28 libraries is shown in Supplementary Table S1. Subsequently, we generated a count table by combining the unique sequences and read counts from all samples, obtaining approximately 5,500,000 unique sequences. Following CPM filtering, a total of 14,200 unique sequences were obtained. We mapped 3844 sequences to Bos taurus mature miRNAs in the miRBase database (v22.1). All isomiRs were grouped under the canonical form to increase the biological relevance of our findings. The isomiR clustering resulted in 132 unique mature miRNAs (Supplementary Table S2).

### 2.4. Cow Individual Variability and Most Abundant miRNAs in Milk EVs in Healthy Quarters

**Figure 2:**
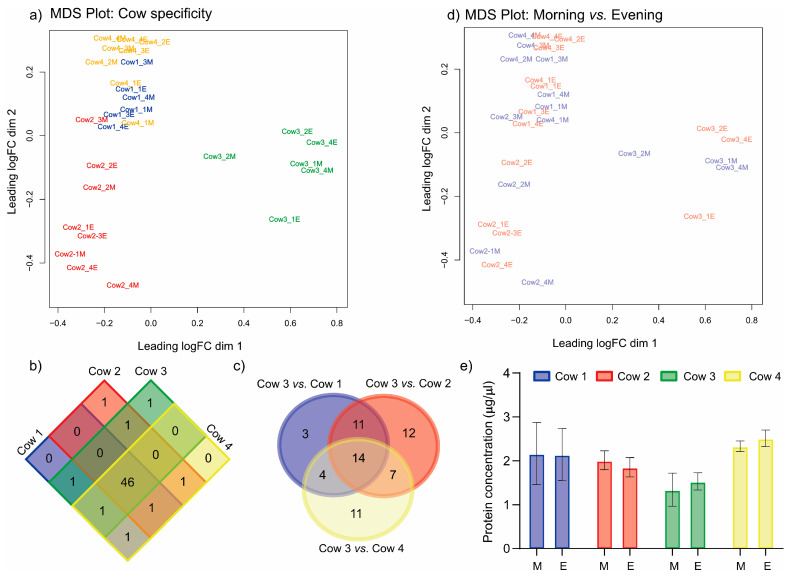
(**a**) Multi-dimensional scaling (MDS) plot of the miRNA profile where each cow is represented by a different color; (**b**) Venn diagram displaying the intersection of the top 50 most abundant miRNAs; (**c**) Venn diagram showing the overlap of altered miRNAs between the three cows; (**d**) MDS plot of the miRNA profile, where morning and evening samples are represented in a different color. The number indicates the collection day (1–4). Mornings (M) are highlighted in blue, and evenings (E) are highlighted in red. (**e**) EV protein concentration for each cow in the morning (M, *n* = 4) and evening (E, *n* = 4) samples. Bar charts show the mean and the standard deviation.

The multidimensional scaling (MDS) plot showed a strong cow influence on the miRNA profile of the milk EVs (Figure 2a). However, the four cows shared 46 of the top 50 most abundant milk EV miRNAs (Figure 2b, Supplementary Table S3). These miRNAs were also observed as the most abundant in our previous experiment [17] and the most common in mammalian milk EVs [27]. Thus, the most abundant miRNAs in milk EVs might have a specific function beyond the pathological status of the mammary gland or other individual cow characteristics. The different miRNAs between Cow 3, the most different one, and the rest of the cows are only partially shared (Figure 2c, Supplementary Table S4).

### 2.5. No Effect of SCC Fluctuations or Circadian Rhythm in miRNA Cargo of Healthy Quarters

We observed high variability in the milk SCC between morning and evening milking sessions, with two cows exhibiting a high SCC value exceeding 200,000 cells/mL despite the absence of any clinical illness (Supplementary Table S1, Supplementary Figure S1). However, this fluctuation was not observed when analyzing the miRNA content of milk EVs (Figure 2d). This result differs from what has been observed in humans, where hsa-miR-16-5p changes between morning and evening [24]. Although the milk EV protein showed no difference between morning and evening, there was a cow effect (*p* = 0.003, Figure 2e). Thus, the EV miRNA profile and protein amounts seem cow-specific, as occurs in other milk components [3]. A recent experiment also observed this result, where healthy cows showed an individual-specific miRNA cargo profile and EV concentration [17].

## 3. Materials and Methods

### 3.1. Animal Ethics, Milk Collection, and SCC Measurement

The animal experiments adhered to the regulations outlined in Swiss law concerning the ethical treatment of animals used for scientific purposes. Additionally, the study received approval from the Committee of Animal Experiments, Canton of Zurich (Switzerland) (ZH/089/18). The experiment was performed with the experimental dairy cow herd at AgroVet Strickhof (Lindau, Switzerland). Only cows showing a negative California Mastitis Test (CMT) and an SCC lower than 100,000 cells/mL were included in the trial. Before sample collection, teats were well cleaned with a wet towel, and the first 5 mL of the foremilk was discarded. Then, 50 mL of milk was collected manually during the morning and evening milking routine for four consecutive days. The SCC value was determined immediately after collection using the DCC DeLaval machine (DeLaval, Sursee, Switzerland). We removed milk fat, cells, and large vesicles by centrifuging the milk at 3000× *g* for 15 min at 4 °C and 12,000× *g* for 20 min at 4 °C. Then, we store the skimmed milk at −20 °C until further use.

### 3.2. Extracellular Vesicles Isolation

Milk EVs were isolated from skim milk by filtering and ultracentrifugation, as previously performed [17,28]. Briefly, 250 µL of acetic acid was mixed with 25 mL of skim milk previously heated at 37 °C for 10 min. Samples were centrifuged at 10,000× *g* for 10 min at 4 °C to precipitate and remove the casein micelles. The first ultracentrifugation (Optimax 90XE, Beckman Coulter, Nyon, Switzerland) was performed after 0.22 μm filtration of the supernatant for 70 min at 210,000× *g* (4 °C). After a PBS wash of the pellet, ultracentrifugation was performed again using the same conditions. Resuspension of the EV pellet was carried out in 500 µL of PBS. Before aliquoting and storing the samples at −80 °C, a last centrifuge of 10,000× *g* for 5 min at 4 °C was carried out.

### 3.3. Extracellular Vesicle Characterization

EV visualization and characterization were achieved using transmission electron microscopy (TEM), tunable resistive pulse sensing (TRPS), and western blot (WB).

#### 3.3.1. TEM

Extracellular vesicles were visualized using the Scientific Center for Optical and Electron Microscopy (ScopEM) service of ETH Zurich, as previously described [17]. For this, 3 µL of the samples were negative-contrast stained and placed on glow-discharged carbon-coated grids. A TEM Morgagni 268 microscope (Thermo Fisher, Zug, Switzerland) operated at 100 kV was used to image the milk EVs. For the immunogold staining, 10 µL of EVs were placed on the grid for 15 min. After two washes in PBS, milk EVs were incubated with the anti-TSG101 primary antibody (50 µg/mL) for 45 min, washed three times with PBS, and incubated with the Au-labeled secondary antibody (10 µg/mL) for 45 min. After three washes with PBS, milk EVs were fixed with 2.5% glutaraldehyde in PBS for 20 min and proceeded with the negative staining of the TEM imaging protocol.

#### 3.3.2. TRPS

The qNano Gold system (Izon Science Ltd., Christchurch, New Zealand) was used to evaluate the particle concentration and diameter, as previously described [17]. Measurements were conducted on diluted milk EV samples (1:100 in filtered PBS) using the NP150 Nanopore and CPC100 calibration beads.

#### 3.3.3. WB

The milk EV pellet was incubated for 20 min (4 °C, 2000 rpm) in radioimmunoprecipitation assay buffer (RIPA buffer, Thermo Fisher) combined with an anti-protease cocktail (Thermo Fisher) to lyse the EVs and extract the proteins. To perform the Western Blots, 10 μg of protein was mixed with 5 μL Laemmli Buffer (Bio-Rad Laboratories, Cressier, Switzerland) and 10% β-Mercaptoethanol if reductant conditions were necessary. After 10 min of incubation at 95 °C, samples were run in a 4–20% Mini-Protein TGX Stain-free Precast gel (Bio-Rad Laboratories) for 30 min at 200 V. The transfer was performed using the 0.2 μm PVDF trans-blot turbo transfer pack (Bio-Rad Laboratories) and the Turbotransfer (Bio-Rad Laboratories) device, with 1.3 A, 25 V, and 7 min as transfer conditions. Membrane blocking was conducted with 5% skim milk powder in TBS-T (TBS buffer, Bio-Rad Laboratories, with 0.05% Tween 20) for 1 h at room temperature. Incubation with the primary antibodies was conducted overnight at 4 °C. The primary antibodies employed were rabbit anti-TSG101 (Thermo Fisher, PA531260, 1:1000), rabbit anti-Calnexin (Abcam, Amsterdam, Netherlands, ab75801, 1:2000), mouse anti-CD81 (Santa Cruz Biotechnology, Dallas, TX, USA, sc166029, 1:300), and mouse anti-CD9 (MCA469GA, Bio-Rad Laboratories, 1:1000). Non-reductant conditions in the protein electrophoresis were used for CD81 and CD9. The secondary antibodies used were goat anti-rabbit IgG-HRF (Santa Cruz Biotechnology, sc2004, 1:10,000) and goat anti-mouse IgM-HRF (Santa Cruz Biotechnology, sc2005 1:10,000). The precision protein Strep Tactin-HRP was also included (1:10,000, Bio-Rad Laboratories) to visualize the ladder. Band visualization was performed using the ChemiDocTM MP and the ClarityTM ECL kit (Bio-Rad Laboratories). A sample of mammary gland tissue was also included in the gel to provide a positive control for calnexin.

### 3.4. Protein Concentration Measurement and Data Analysis

Protein quantification was performed with the BCA protein assay kit (Thermo Fisher) and the Nanodrop 2000 (Thermo Fisher). Statistical analyses were performed using GraphPad Prism 8.2 software. The normality of the EV protein concentration was assessed using a Shapiro-Wilk test. A multiple linear regression test was performed to analyze EV protein concentration, including time (morning or evening) and cow in the model. Differences were considered significant if *p* < 0.05.

### 3.5. RNA Extraction, Small RNA Library Preparation, and Data Analysis

Total RNA was extracted and measured from 200 μL of milk EVs using the miRNeasy Micro Kit (Qiagen, Hombrechtikon, Switzerland) [17]. Afterward, the Quantus™ Fluorometer and the QuantiFluor^®^ RNA System kit (Promega, Dübendorf, Switzerland) were used to determine the RNA quantity. We used the NEXTflex™ Small RNA-Seq Kit v3 (Bioo Scientific, Austin, TX, USA) to generate libraries starting from 10 ng of total RNA per sample. Library sequencing was performed at the Functional Genomic Centre Zurich (FGCZ) using an Illumina HiSeq2500 (126 bp single-end reads). We cleaned, filtered, and annotated the sequences with the in-house developed pipeline on our local Galaxy system described before [17]. We applied the count per million (CPM) cut-off at 8.29, corresponding to an average of >10 reads per sample for at least 6 of the 28 libraries. We performed differential expression analysis using the Bioconductor package EdgeR [29], and differences with an adjusted *p*-value (false discovery rate FDR) <5% were considered significant. We deposited the small RNA-seq data in the Gene Expression Omnibus database (GEO) under accession code GSE232617.

## 4. Conclusions

Our findings affirm the consistent stability of milk EV miRNAs, irrespective of SCC variations during health and the time of the day, and highlight the potential of milk EV miRNAs as valuable diagnostic and prognostic markers for assessing mammary gland health.

## Data Availability

Small RNA-seq data have been deposited in the Gene Expression Omnibus database (GEO) under accession code GSE232617.

## Supplementary Materials

The supporting information can be downloaded at: https://www.mdpi.com/article/10.3390/ijms241210210/s1.
