# Supplementary material for: Circadian Rhythm Does Not Affect the miRNA Cargo of Bovine Raw Milk Extracellular Vesicles
Source: Int J Mol Sci. 2023 Jun 16;24(12):10210. doi: 10.3390/ijms241210210 (PMC10299634; doi:10.3390/ijms241210210)
Supplement: Supplementary file 1 [file ijms-24-10210-s001.zip › Supplementary Figure S1 - Saenz de Juano 2023.pdf]

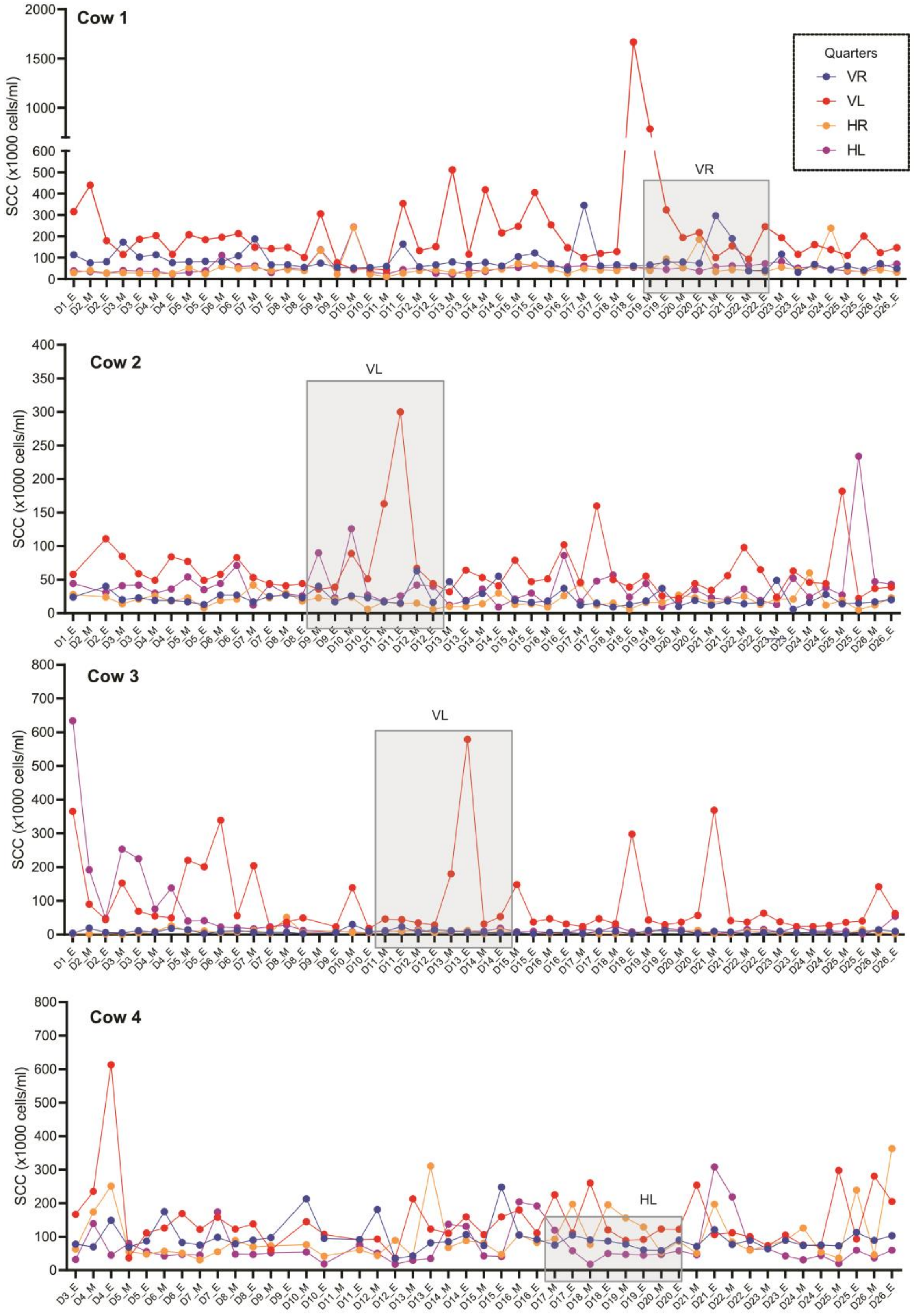

Figure S1. Somatic Cell Count (SCC) of each cow and each quarter values during the morning (M) and Evening (E) for 26 consecutive day
